# Maternal diabetes subtypes and offspring cutaneous health: developmental programming of sebaceous gland function in over 330,000 live births

**DOI:** 10.1007/s00404-026-08398-z

**Published:** 2026-05-15

**Authors:** Eliya Honig, Nir Amitai, Sarah Weissmann, Amir Horev, Tamar Eshkoli

**Affiliations:** 1 Department of Obstetrics and Gynecology, Soroka University Medical Center, Yitzhak Rager Ave., POB 151, 8410101 Beer-Sheva, Israel; 2 Faculty of Health Sciences, Ben-Gurion University of the Negev, Beer Sheva, Israel; 3 Clinical Research Unit, Soroka University Medical Center, Beer Sheva, Israel; 4 Pediatric Dermatology Service, Soroka University Medical Center, Beer Sheva, Israel; 5 Endocrinology Unit, Soroka University Medical Center, Beer Sheva, Israel

**Keywords:** Gestational diabetes mellitus, Seborrheic dermatitis, Fetal programming, Intrauterine environment, Metabolic imprinting

## Abstract

**Objective:**

Maternal diabetes is a known driver of fetal metabolic programming, yet its impact on offspring cutaneous health remains poorly characterized. We investigated the impact of intrauterine exposure to various maternal diabetes subtypes on the risk of offspring seborrheic dermatitis (SD), and to explore whether this risk is modulated by maternal glycemic control or treatment modality.

**Methods:**

This large-scale, population-based cohort study included 331,335 mother–child pairs. Maternal diabetes subtypes—type 1 (T1DM), type 2 (T2DM), and gestational diabetes mellitus (GDM)—were assessed, along with glycemic control (HbA1c) and pharmacological treatment. SD was identified using physician-documented diagnoses and medication records. Multivariable logistic regression models adjusted for maternal and perinatal factors were used to estimate adjusted odds ratios (aORs).

**Results:**

The incidence of SD was 4.5%. SD was significantly more frequent among offspring of mothers with diabetes (4.9% vs. 4.4%, p < 0.001), primarily driven by GDM. T1DM and T2DM showed similar trends but weren't statistically significant. SD risk did not differ by treatment modality or glycemic control. While overall associations for T1DM and T2DM did not reach significance in the full cohort, age-stratified analysis revealed that maternal diabetes was significantly associated with increased SD risk across all subtypes within the first year of life (T1DM: p = 0.033, T2DM: p = 0.024, GDM: p = 0.019). Multivariable analysis showed maternal diabetes was independently associated with increased SD risk (aOR 1.16, 95% CI 1.08–1.24).

**Conclusion:**

Maternal diabetes is associated with a significantly increased risk of infantile SD across all diabetes subtypes, suggesting that the intrauterine diabetic environment may influence early-life cutaneous homeostasis through metabolic programming of the pilosebaceous unit.

**Supplementary Information:**

The online version contains supplementary material available at 10.1007/s00404-026-08398-z.

## What does this study add to the clinical work

**Table:** 

Maternal diabetes is associated with an increased risk of infantile seborrheic dermatitis. This risk is most pronounced during the first year of life across all maternal diabetes subtypes, suggesting a time-limited ‘metabolic priming’ effect on the fetal pilosebaceous unit’.	

## Introduction

Seborrheic dermatitis (SD) is a prevalent chronic inflammatory skin disorder, affecting sebaceous gland-rich regions. Its global prevalence follows a bimodal distribution, with a significant peak in infants under three months and another during adolescence [1–3]. Beyond its physical symptoms, the visible nature of SD—characterized by erythema and flaking—can lead to psychological distress and significantly impact the quality of life for both affected individuals and their families.

The pathogenesis of SD is multifactorial, involving a complex interplay between sebocyte activity, skin microbiome dysbiosis (particularly *Malassezia* species), genetic predisposition, and immune dysregulation [2, 4–7]. Recent advances highlight the critical roles of immune-mediated inflammation and skin barrier dysfunction in the onset of the disease [4, 8–11]. Notably, infantile SD emerges during a period of rapid maturation of the skin barrier and innate immune system. Given that fetal sebaceous glands are highly sensitive to maternal hormonal and metabolic signals, early-life metabolic alterations—such as those associated with maternal diabetes—may fundamentally influence disease susceptibility.​

Accumulating evidence suggests that maternal diabetes during pregnancy exerts a long-term influence on offspring health through fetal immune and metabolic programming [12–22]. While maternal diabetes has been linked to an increased risk of other inflammatory skin conditions, such as atopic dermatitis [16, 23], its association with offspring SD remains unexplored. The objective of this study is to elucidate the association between pregestational and gestational maternal diabetes and the risk of SD in offspring, utilizing a large population-based cohort to address this significant knowledge gap.

## Materials and methods

### Data source

This retrospective cohort study was conducted using the Clalit Health Services (CHS) data-sharing platform, powered by MDClone (https://www.mdclone.com). The platform employs advanced algorithms to extract and de-identify data from electronic medical records, thereby ensuring both patient privacy and data integrity. The study protocol was approved by the Ethics Committee of Soroka University Medical Center (approval No. 0049–25-SOR).

### Study population

CHS is the largest health organization in Israel, providing care for approximately 4.8 million members—about 51% of the national population. The study population included all live births recorded between January 1, 2010, and December 31, 2023. Children were followed until December 31, 2024, to guarantee at least one year of follow-up for each child.

Eligibility required both the infant and mother to be CHS members during the study period. The initial dataset contained 352,299 live births. After excluding 20,964 multiple gestations, the final analytic sample consisted of 331,335 singleton births.

### Variable definitions

The principal exposure of interest was maternal diabetes during pregnancy, defined according to International Classification of Diseases, 10th Revision (ICD-10) codes documented prior to delivery or by a recorded prescription for anti-diabetic medication before delivery. The diabetes-exposed group was subsequently categorized into type 1 diabetes (T1DM), type 2 diabetes (T2DM), and gestational diabetes mellitus (GDM) based on ICD diagnoses recorded in the medical file. The primary study outcome was a diagnosis of SD in offspring, identified through ICD-10 codes available in the CHS database. Diagnoses were recorded by board-certified pediatricians, family physicians, or dermatologists during outpatient or inpatient encounters. To ensure clinical relevance, cases were identified based on physician-documented diagnoses. In the context of the CHS database, such coding reflects a clinical consultation where the severity of the condition warranted medical attention, often resulting in relevant prescriptions for topical treatments.

Maternal prescriptions for anti-diabetic medication were identified through Anatomical Therapeutic Chemical (ATC) classification system codes at the fifth level (ATC-5), capturing prescriptions dispensed within the nine months preceding delivery.

Additional maternal and perinatal covariates included maternal age at delivery, maternal smoking status, maternal history of allergic rhinitis, and maternal glycated hemoglobin (HbA1C) levels measured between 18 and 6 months prior to delivery as indicators of glycemic control and proxies for disease severity. Pregnancy and infant related covariates included mode of delivery, gestational age, infant birth weight, child sex, SES, and follow-up duration. SES was derived from a municipality-level index developed by the Israeli Central Bureau of Statistics, based on 14 socioeconomic indicators. Municipalities were classified into deciles (1–10) and further grouped into low (1–3), medium (4–6), and high (7–10) categories.

### Statistical analysis

Descriptive statistics summarized baseline maternal and infant characteristics. Continuous variables are reported as means, medians, standard deviations (S.D.), interquartile ranges (IQR), and ranges, while categorical variables are presented as counts and percentages. Comparisons of continuous variables were performed using Student’s t-test when normally distributed and the Mann–Whitney U test otherwise. Proportions were compared using Chi-square tests, with Fisher’s exact test applied in cases of sparse data.

Logistic regression models were used to evaluate associations between maternal diabetes during pregnancy and S.D., with results expressed as odds ratios (ORs) and 95% confidence intervals (CIs). Multivariable models adjusted for child sex, maternal age, SES, ethnicity, gestational age at birth, maternal smoking status, and follow-up duration.

All analyses were two-sided, and statistical significance was defined as P < 0.05. Data processing and analyses were performed using R software, version 4.2.3.

## Results

The cohort included a total of 331,335 mother–child pairs, comprising 310,600 offspring to mothers without diabetes and 20,735 offspring to mothers with any type of diabetes. Clinical and demographic characteristics of the participants are listed in Table 1.

**Table 1:** Patient characteristics

Characteristic	Overall N = 331,335	Without diabetes N = 310,600 [Reference]	With any diabetes N = 20,735	p-value
Maternal age at delivery				< 0.001
Mean ± SD	30.3 ± 5.6	30.1 ± 5.5	33.5 ± 5.2	
Median (IQR)	30.3 (26.2–34.3)	30.1 (26.0–34.1)	33.6 (29.8–37.2)	
Range	15.2–50.0	15.2–50.0	16.4–50.0	
Maternal parity,				
Mean ± SD	1.68 ± 1.77	1.66 ± 1.76	1.95 ± 1.92	< 0.001
Gestational week				< 0.001
Mean ± SD	38.89 ± 1.72	38.93 ± 1.71	38.25 ± 1.66	
Median (IQR)	39.00 (38.00–40.00)	39.00 (38.00–40.00)	38.00 (38.00–39.00)	
Range	24.00–44.00	24.00–44.00	24.00–44.00	
Preterm birth (< 36 weeks), n (%)	19,305 (5.8%)	17,501 (5.6%)	1804 (8.7%)	< 0.001
Delivery type, n (%)				< 0.001
Cesarean	50,568 (17%)	45,056 (17%)	5512 (31%)	
Vaginal	239,960 (83%)	227,633 (83%)	12,327 (69%)	
Maternal allergic rhinitis, n (%)	43,588 (13%)	40,309 (13%)	3279 (16%)	< 0.001
Maternal atopic dermatitis, n (%)	28,394 (8.6%)	26,458 (8.5%)	1936 (9.3%)	< 0.001
Maternal other atopic state, n (%)	65,551 (20%)	60,843 (20%)	4708 (23%)	< 0.001
Maternal smoking status, n (%)				< 0.001
Non-smoker	294,953 (89%)	277,395 (90%)	17,558 (85%)	
Past smoker	4,046 (1.2%)	3,694 (1.2%)	352 (1.7%)	
Current smoker	31,238 (9.5%)	28,439 (9.2%)	2799 (14%)	
Ethnicity, n (%)				< 0.001
Arabic	36,552 (11%)	34,478 (11%)	2074 (10%)	
Bedouin	64,517 (20%)	62,061 (20%)	2456 (12%)	
Jewish	225,034 (69%)	209,194 (68%)	15,840 (78%)	
Socioeconomic score, n (%)				< 0.001
Low	91,960 (31%)	87,690 (32%)	4270 (22%)	
Medium	150,852 (51%)	139,774 (50%)	11,078 (58%)	
High	53,435 (18%)	49,694 (18%)	3741 (20%)	
Offspring follow-up duration				< 0.001
Mean ± SD	6.1 ± 3.3	6.2 ± 3.3	5.5 ± 3.2	
Median (IQR)	6.0 (3.0–9.0)	6.0 (3.0–9.0)	5.0 (3.0–8.0)	
Range	1.0–14.0	1.0–14.0	1.0–14.0	
Offspring gender, n (%)				0.004
Female	160,855 (49%)	150,992 (49%)	9863 (48%)	
Male	170,477 (51%)	159,605 (51%)	10,872 (52%)	

*SD* Standard deviation; *IQR* Interquartile range

Reference category: Without diabetes (N = 310,600). p-values represent comparison between with-diabetes and without-diabetes groups

The overall incidence of SD in the study population was 4.5%. Children born to mothers with any type of diabetes had an incidence of 4.9%, compared to 4.4% in offspring of mothers without diabetes (p = 0.003) (Table 2). This corresponds to an absolute risk increase (ARI) of 0.44% and a number needed to harm (NNH) of 229. Among the different types of maternal diabetes, multivariable analysis showed that T2DM exhibited the strongest association with offspring SD (aOR 1.38, 95% CI 1.17–1.62, p < 0.001), followed by GDM (aOR 1.11, 95% CI 1.02–1.20, p = 0.013). Crucially, when stratified by age at diagnosis, the association was driven entirely by infantile SD (diagnosed at < 1 year of age), where any maternal diabetes was associated with a significantly higher risk (3.2% vs. 2.8%, p < 0.001). This trend was consistent across all subtypes, including T1DM (3.8% vs 2.8%, p = 0.033), T2DM (3.4% vs 2.8%, p = 0.024), and GDM (3.1% vs 2.8%, p = 0.019). In contrast, no significant association was observed for SD diagnosed after one year of age (1.7% vs. 1.7%, p = 0.7). The mean age at SD diagnosis was similar across all groups (1.80 ± 2.64 years for diabetes-negative vs. 1.64 ± 2.46 years for any diabetes; p = 0.6).

**Table 2:** Incidence and Timing of Offspring Seborrheic Dermatitis by Maternal Diabetes Status

Characteristic	Diabetes-Negative (N = 310,600) [Reference]	Any Diabetes Positive (N = 20,735)	p-value
Seborrheic dermatitis diagnosis, n (%)	13,789 (4.4%)	1011 (4.9%)	0.003
SD before age 1 year, n (%)	8553 (2.8%)	655 (3.2%)	< 0.001
SD after age 1 year, n (%)	5236 (1.7%)	356 (1.7%)	0.7
Age at SD diagnosis (years)			0.6
Mean ± SD	1.80 ± 2.64	1.64 ± 2.46	
Median (IQR)	0.44 (0.19–2.38)	0.43 (0.21–2.21)	
Range	0.00–14.77	0.04–14.63	

*SD* Seborrheic dermatitis; *IQR* Interquartile range

Reference category: Diabetes-negative children (N = 310,600). Breakdown by diabetes subtype (T1DM, T2DM, GDM) is presented in Supplementary Table S1

Detailed breakdown of SD incidence by maternal diabetes subtype is provided in Supplementary Table S1*.*

When SD incidence was examined according to maternal treatment modality (Table 3), no significant differences were observed. Children of mothers who did not receive pharmacological treatment had an incidence of 4.9%, compared to 5.0% for insulin-treated mothers, 4.5% for those treated with sulfonylurea, and 4.4% for metformin-treated mothers (all p > 0.2). The mean age at diagnosis of SD was also comparable across all groups, with no significant differences observed.

**Table 3:** Offspring morbidity by diabetic treatment

Characteristic	Overall N = 20,735			Insulin treatment			Sulfonylurea treatment			Metformin treatment		
	Lifestyle changes N = 14,729	Pharmacologic N = 6006	p-value	Negative [Ref] N = 16,728	Positive N = 4007	p-value	Negative [Ref] N = 19,829	Positive N = 906	p-value	Negative [Ref] N = 17,937	Positive N = 2798	p-value
Seborrheic dermatitis diagnosis, n (%)	721 (4.9%)	290 (4.8%)	0.8	809 (4.8%)	202 (5.0%)	0.6	970 (4.9%)	41 (4.5%)	0.6	887 (4.9%)	124 (4.4%)	0.2
SD before age 1 year, n (%)	461 (3.1%)	194 (3.2%)	0.7	524 (3.1%)	131 (3.3%)	0.7	630 (3.2%)	25 (2.8%)	0.5	563 (3.1%)	92 (3.3%)	0.7
SD after age 1 year, n (%)	260 (1.8%)	96 (1.6%)	0.4	285 (1.7%)	71 (1.8%)	0.8	340 (1.7%)	16 (1.8%)	> 0.9	324 (1.8%)	32 (1.1%)	0.012
Age at SD diagnosis (years)			0.5			0.8			0.2			0.028
Mean ± SD	1.67 ± 2.45	1.54 ± 2.48		1.62 ± 2.39	1.70 ± 2.72		1.63 ± 2.46	1.86 ± 2.48		1.73 ± 2.56	0.98 ± 1.43	
Median (IQR)	0.43 (0.21–2.31)	0.41 (0.20–1.91)		0.42 (0.21–2.29)	0.43 (0.22–1.94)		0.42 (0.20–2.18)	0.60 (0.27–3.02)		0.43 (0.21–2.34)	0.35 (0.19–1.05)	
Range	0.04–12.69	0.07–14.63		0.04–12.69	0.07–14.63		0.04–14.63	0.08–10.41		0.04–14.63	0.09–8.74	

*SD* Seborrheic dermatitis; *IQR* Interquartile range; *Ref* Reference category

Reference categories: Insulin-negative (N = 16,728); Sulfonylurea-negative (N = 19,829); Metformin-negative (N = 17,937). p-values represent comparison between treatment-positive and treatment-negative groups within the diabetes-positive cohort

SD incidence by maternal glycemic control (HbA1c < 6.5 vs. ≥ 6.5) is shown in Table 4. Among offspring of mothers with HbA1c < 6.5, the incidence was 4.4% (1,664/37,723), whereas among offspring of mothers with HbA1c ≥ 6.5, the incidence was 5.0% (76/1,527; p = 0.3).

**Table 4:** Offspring morbidity by maternal glycemic control (HbA1c > 6.5%)

Characteristic	HbA1c < 6.5% (N = 37,723) [Reference]	HbA1c > 6.5% (N = 1,527)	p-value
Seborrheic dermatitis diagnosis, n (%)	1664 (4.4%)	76 (5.0%)	0.3
SD before age 1 year, n (%)	1107 (2.9%)	54 (3.5%)	0.2
SD after age 1 year, n (%)	557 (1.5%)	22 (1.4%)	> 0.9
Age at SD diagnosis (years)			0.7
Mean ± SD	1.51 ± 2.33	1.40 ± 2.51	
Median (IQR)	0.38 (0.18–1.85)	0.38 (0.19–1.32)	
Range	0.03–12.66	0.09–14.39	

*SD* Seborrheic dermatitis; *IQR* Interquartile range

Reference category: HbA1c < 6.5% (N = 37,723). Overall N = 39,250

Results of the multivariate logistic regression analysis are presented in Table 5. After adjustment for child gender, maternal age, gestational age, socioeconomic status, ethnicity, allergic rhinitis, maternal smoking, and child follow-up duration, maternal diabetes was associated with an increased risk of SD in offspring. The crude odds ratio (OR) was 1.10 (95% CI 1.03–1.18; p = 0.003), and the adjusted OR was 1.16 (95% CI 1.08–1.24; p < 0.001).

**Table 5:** Multivariate regression: association between maternal diabetes and offspring seborrheic dermatitis

Exposure	Crude OR	95% CI	p-value	Adjusted OR	95% CI	p-value
Any maternal diabetes	1.10	1.03–1.18	0.003	1.16	1.08–1.24	< 0.001
Maternal T1DM	1.14	0.87–1.49	0.327	1.15	0.86–1.53	0.353
Maternal T2DM	1.19	1.03–1.38	0.022	1.38	1.17–1.62	< 0.001
Maternal GDM	1.08	1.00–1.16	0.051	1.11	1.02–1.20	0.013
Reference: No maternal diabetes	Ref	—	—	Ref	—	—

*OR* Odds ratio; *CI* Confidence interval; *Ref* Reference category

Reference category: No maternal diabetes (N = 310,600)

^*^Adjusted for: offspring gender, maternal age, gestational age, socioeconomic scale, ethnicity, allergic rhinitis, maternal smoking status, child follow-up duration

## Discussion:

In this large population-based study, we found that maternal diabetes mellitus was associated with a modest but statistically significant increase in the risk of infantile SD, a finding consistent across all diabetes subtypes in the first year of life. While the absolute risk difference was small (ARI 0.44%, NNH 229), the association was specifically localized to the first year of life.

Crucially, our age-stratified analysis (Table 2) demonstrated that this risk was strictly confined to infancy. While the incidence of SD before age 1 was significantly higher among offspring of mothers with diabetes (3.2% vs. 2.8%, p < 0.001), no such difference was observed for diagnoses made after the first year. This temporal specificity—where the risk dissipates as the child’s own metabolic milieu matures—is highly suggestive of an intrauterine 'metabolic priming' effect rather than shared genetic or postnatal environmental factors.

The pathogenesis of SD is multifactorial, involving an interplay between sebocyte activity, skin microbiome dysbiosis (particularly *Malassezia* species) and immune dysregulation [4–11]. We hypothesize that fetal hyperinsulinemia—a hallmark of maternal diabetes—acts as a growth stimulus for neonatal sebaceous glands. Insulin and IGF-1 receptors are highly expressed in human sebocytes, stimulating cellular proliferation and lipogenesis. This in-utero priming may lead to transient sebaceous gland hyperplasia and altered sebum production, providing a favorable substrate for *Malassezia* colonization and the subsequent inflammatory responses characteristic of SD [24]. Furthermore, maternal diabetes is known to induce broader fetal immune dysregulation and microbial imprinting [25–32]. Infants born to mothers with GDM often exhibit reduced microbial diversity and pro-inflammatory signatures, which could further increase vulnerability to the inflammatory cascades involved in SD, such as NLRP3 inflammasome activation [25].

These observations align with the Developmental Origins of Health and Disease (DOHaD) framework [28], which posits that intrauterine exposures can fundamentally re-program neonatal physiological systems. Our findings mirror evidence linking maternal diabetes to other inflammatory conditions, such as atopic dermatitis [16, 23]. Furthermore, previous large-scale studies from our center have demonstrated that intrauterine exposure to maternal diabetes is associated with a wide spectrum of long-term offspring morbidities, including respiratory, neuropsychiatric, and endocrine disorders [20–22]. Since these conditions share overlapping mechanisms of skin barrier impairment and immune dysregulation, the elevated SD risk likely reflects a broader impact of maternal metabolic status on early-life cutaneous health.

Notably, while significant associations emerged for all subtypes in the age-stratified analysis, GDM exhibited the most robust association in our adjusted models. This may be attributed to the timing of the metabolic insult; GDM typically manifests during the third trimester, coinciding precisely with the peak development of fetal sebaceous glands. The lack of association with maternal glycemic control (HbA1c) or treatment modality may suggest a 'threshold effect,' where the presence of a diabetic environment itself triggers the priming effect. Although HbA1c was assessed 6–18 months prior to delivery to maximize data coverage, these baseline values may not capture the transient postprandial glucose spikes critical to fetal skin development.

## Strengths, limitations, and future directions

To our knowledge, this is the first large-scale population-based study to evaluate the association between maternal diabetes and seborrheic dermatitis in offspring. While prior studies have focused on metabolic and allergic outcomes, cutaneous manifestations have received relatively little attention in this context. The use of a large, non-selected national cohort minimizes referral bias and ensures that our findings are representative of the general population. The study benefits from a large sample size, comprehensive adjustment for potential confounders, and rigorous outcome assessment. Additionally, it utilizes long-term data from a unified health maintenance organization (HMO) database, ensuring reliable coding and consistent data quality over time. The ability to distinguish between types of diabetes further strengthens the study by allowing detailed subgroup analyses.

However, several limitations exist: First, SD was identified via physician-recorded ICD-10 codes. While some diagnostic overlap with physiologic cradle cap is possible, our reliance on physician-documented coding serves as an inherent clinical filter, as such coding is linked to a formal medical encounter. This ensures that even in infancy, the cases identified were clinically significant enough to warrant medical consultation and were not merely transient symptoms. Furthermore, any potential misclassification is expected to be non-differential, which typically biases results toward the null, suggesting our findings may be conservative estimates of the true biological effect.

Second, while the absolute risk difference was small (ARI 0.44%), it reflects a meaningful population-level impact given the high prevalence of both conditions.

Third, the relatively small sample size for pre-gestational diabetes, particularly T1DM (n = 1104), may have limited our statistical power in the non-stratified analysis. The wider confidence intervals in these subgroups reflect lower statistical precision rather than a definitive absence of biological effect. However, the emergence of significant associations across all diabetes types in our age-stratified model (SD before age 1) suggests a consistent biological trend. We therefore emphasize that our results represent a conservative estimate, where the degree of uncertainty in specific point estimates is largely a function of the available sample size in those strata.

Fourth, our reliance on HbA1c values from 6–18 months prior to delivery may introduce non-differential misclassification. This window was chosen to maximize data availability, as HbA1c is more consistently recorded during the pre-conception and early pregnancy periods compared to later stages. Since glycemic control fluctuates, this lack of proximal data likely biased our findings toward the null, potentially underestimating the true association.

Finally, data on residual confounders such as breastfeeding, infant skin care practices, and antibiotic exposure were unavailable. While these postnatal factors influence the neonatal microbiome and skin barrier, the 'metabolic priming' of the fetal pilosebaceous unit—driven by maternal hyperglycemia and fetal hyperinsulinemia—occurs in utero, prior to these exposures. Thus, while unmeasured factors like dysbiosis may modulate SD severity, they are unlikely to fully account for the robust association observed during the infantile peak of the disease. Nevertheless, future prospective studies should incorporate these variables to better isolate the independent effect of maternal diabetes.

## Conclusion

In summary, our findings demonstrate that maternal diabetes, particularly gestational diabetes, is associated with a small but statistically significant increase in the risk of infantile seborrheic dermatitis. These results support the Developmental Origins of Health and Disease (DOHaD) framework [25], suggesting that the intrauterine diabetic environment may induce lasting alterations in the offspring's cutaneous metabolic and inflammatory pathways. While the absolute risk difference is modest, this study highlights the fetal skin as a relevant target for developmental imprinting. These findings underscore the importance of a holistic approach to prenatal care and suggest that the long-term dermatological health of the offspring may be influenced by maternal metabolic status during critical gestational windows.

## Supplementary Information

Below is the link to the electronic supplementary material.

Supplementary file1 (DOCX 29 KB)

## Data Availability

The data that support the findings of this study are available from the corresponding author upon reasonable request, subject to the approval of Clalit Health Services and the relevant Institutional Review Board.
