# Supplementary material for: Maternal diabetes subtypes and offspring cutaneous health: developmental programming of sebaceous gland function in over 330,000 live births
Source: Arch Gynecol Obstet. 2026 May 15;313(1):219. doi: 10.1007/s00404-026-08398-z (PMC13346271; doi:10.1007/s00404-026-08398-z)
Supplement: Supplementary file 1 — Supplementary file1 (DOCX 29 KB) [file 404_2026_8398_MOESM1_ESM.docx]

**Supplementary Table S1. Offspring morbidity by diabetes subtype (T1DM, T2DM, GDM)**

| **Characteristic** | **Diabetes-Negative (N=310,600) [Reference]** | **T1DM (N=1,104)** | **P** | **T2DM (N=3,623)** | **p** | **GDM (N=16,008)** | **p** |
| --- | --- | --- | --- | --- | --- | --- | --- |
| Seborrheic dermatitis diagnosis, n (%) | 13,789 (4.4%) | 56 (5.1%) | 0.3 | 190 (5.2%) | 0.019 | 765 (4.8%) | 0.042 |
| SD before age 1 year, n (%) | 8,553 (2.8%) | 42 (3.8%) | 0.033 | 122 (3.4%) | 0.024 | 491 (3.1%) | 0.019 |
| SD after age 1 year, n (%) | 5,236 (1.7%) | 14 (1.3%) | 0.3 | 68 (1.9%) | 0.4 | 274 (1.7%) | 0.8 |
| **Age at SD diagnosis (years)** |  |  | 0.081 |  | >0.9 |  | 0.9 |
| Mean ± SD | 1.80 ± 2.64 | 1.23 ± 2.49 |  | 1.75 ± 2.63 |  | 1.64 ± 2.42 |  |
| Median (IQR) | 0.44 (0.19–2.38) | 0.34 (0.18–1.02) |  | 0.47 (0.21–2.12) |  | 0.42 (0.21–2.31) |  |
| Range | 0.00–14.77 | 0.08–14.39 |  | 0.05–10.72 |  | 0.04–14.63 |  |

T1DM = Type 1 diabetes mellitus; T2DM = Type 2 diabetes mellitus; GDM = Gestational diabetes mellitus; SD = Seborrheic dermatitis.

Reference category for all comparisons: Diabetes-negative children (N = 310,600).
